# On the use of catalysis to bias reaction pathways in out-of-equilibrium systems†

**DOI:** 10.1039/d0sc06406h

**Published:** 2021-02-09

**Authors:** Michelle P. van der Helm, Tuanke de Beun, Rienk Eelkema

**Affiliations:** Department of Chemical Engineering, Delft University of Technology Van der Maasweg 9 2629 HZ Delft The Netherlands R.Eelkema@tudelft.nl +31 15 27 81035

## Abstract

Catalysis is an essential function in living systems and provides a way to control complex reaction networks. In natural out-of-equilibrium chemical reaction networks (CRNs) driven by the consumption of chemical fuels, enzymes provide catalytic control over pathway kinetics, giving rise to complex functions. Catalytic regulation of man-made fuel-driven systems is far less common and mostly deals with enzyme catalysis instead of synthetic catalysts. Here, we show *via* simulations, illustrated by literature examples, how any catalyst can be incorporated in a non-equilibrium CRN and what their effect is on the behavior of the system. Alteration of the catalysts' concentrations in batch and flow gives rise to responses in maximum conversion, lifetime (*i.e.* product half-lives and *t*90 – time to recover 90% of the reactant) and steady states. *In situ* up or downregulation of catalysts' levels temporarily changes the product steady state, whereas feedback elements can give unusual concentration profiles as a function of time and self-regulation in a CRN. We show that simulations can be highly effective in predicting CRN behavior. In the future, shifting the focus from enzyme catalysis towards small molecule and metal catalysis in out-of-equilibrium systems can provide us with new reaction networks and enhance their application potential in synthetic materials, overall advancing the design of man-made responsive and interactive systems.

## Abstract 2

We show, *via* simulations, how catalytic control over individual paths in a fuel-driven non-equilibrium chemical reaction network in batch or flow gives rise to responses in maximum conversion, lifetime and steady states.

## Graphical abstract

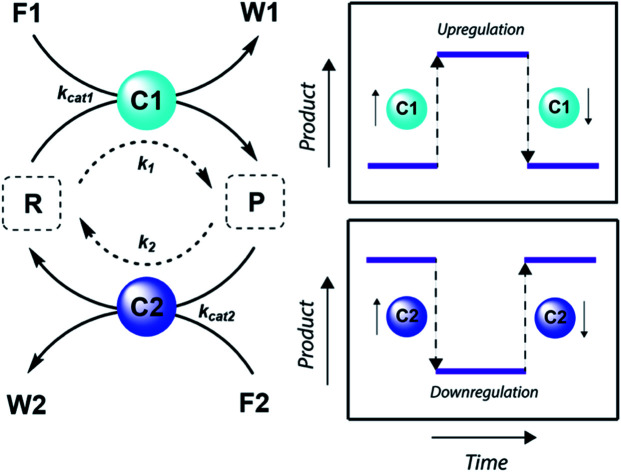

## Introduction

Regulation of chemical processes by catalytic activity is crucial to life. Metabolic pathways rely heavily on enzymes to catalyse individual reaction steps and control complex reaction networks. In contrast, deregulation of enzymes in signal transduction pathways can have severe consequences for cells and their organisms by generation of oncogenic signals and the onset of cancer, *e.g.* for tyrosine kinase in acute myeloid leukemia.^1,2^ Much of the complex functions in living systems are encoded in out-of-equilibrium (bio)chemical reaction networks (CRNs) and require an input of chemical fuel, which is often a high-energy bond molecule, like the phosphoanhydride bond in ATP (adenosine triphosphate) and GTP (guanosine triphosphate).^3–7^ Examples of such fuel-driven systems in biology include the actin and microtubule protein filament assembly which make up the cytoskeleton, histone acetylation to regulate gene transcription and ribosomal protein biosynthesis with aminoacyl-*t*RNA synthetases.^8^ All these biological examples combine a fuel-driven system with catalysis to achieve highly dynamic behaviour and control vital cellular processes.

At the basis of the above systems are out-of-equilibrium CRNs, reaction networks consisting of at least two competing reactions (product formation and degradation). Essential properties of such systems are dictated by reaction kinetics rather than thermodynamic stability and the products can only be sustained in the presence of enough chemical fuel. The transient state of the system will start to disintegrate when the chemical fuel is depleted, whereupon it converts back to the thermodynamic equilibrium state of the starting building blocks. As in the biological examples above, the reaction kinetics of the forward and backward reaction can be regulated by catalysis, which in turn can have a large impact on the behavior of the system. To illustrate this, in this work, we show the kinetic modelling of a generalized fuel-driven system, where catalysts are introduced to alter reaction kinetics, allowing pathway biasing in an out-of-equilibrium system and shifting between out-of-equilibrium steady states. Both batch and flow systems are investigated, as well as feedback mechanisms. The modelling results are illustrated using relevant literature examples of catalytic out-of-equilibrium systems. Furthermore, we conclude this article with directions for future research in this field. Noteworthy, we focus on the impact of catalysis on reaction pathways governing these fuel-driven reaction networks and less on the behaviour, function or application of the transient product. For the latter we refer to a comprehensive review from our group with the Boekhoven group,^6^ two other reviews on application and design from the Boekhoven group,^9,10^ a review from the Klajn group,^11^ a perspective from the Prins group^12^ and a recent review from the Hermans group.^13^

## Catalytic network regulation in fuel-driven batch and flow systems

**Scheme 1:**
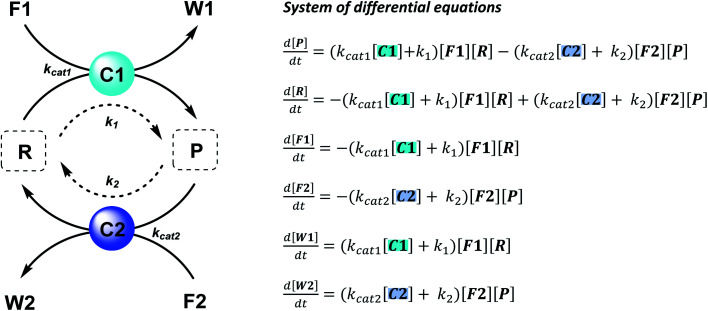
Generic fuel-driven CRN, where both formation and degradation pathways are catalysed. Reactant (R) is converted into product (P) by supplying fuel (F1) and concomitantly generating waste (W1), catalysed by C1. P is unstable and only transiently formed and degrades again with reagent (F2), generating R and waste (W2), catalysed by C2.

The majority of man-made fuel-driven systems makes use of batch-wise fuel additions. A key control factor in these systems is the supply of fuel. The amount of fuel added typically changes the yield and lifetime of the transient product. With lifetime we mean here the duration that the transient product is present in significant amounts, in other words the time it takes to bring the system back to the thermodynamic equilibrium state. This lifetime is directly affected by the presence of fuel. Since lifetime is not easily quantified, we calculate the half-life of the active product and the *t*90, the time it takes to regenerate 90% of the starting material (or have 10% of the product remain). Although batch-wise additions have been mostly studied so far, studying continuous supply of fuel can be very interesting. Continuous chemical fuel supply can give rise to unusual features, like oscillations, bistable switching or chaotic behaviour.^5,14^ Besides the fuel, introducing catalysts in an out-of-equilibrium CRN to alter the reaction kinetics of the forward or backward reaction can provide additional control elements. Numerical simulations of reaction networks are not new,^5,15^ we now apply this concept to elucidate the role of catalysis to bias reaction network paths. Thus, in the current work, we established two kinetic models (which are available as ESI†) based on a minimalistic reaction network (Scheme 1), to be able to compare the influence of catalysis in batch and flow fuel-driven systems. In this CRN, reactant (R) is converted into product (P) by supplying chemical fuel (F1) and concomitantly generating waste (W1). This formation reaction is catalysed by catalyst C1 with rate constant *k*_cat1_ and an uncatalysed background reaction with constant *k*_1_. P is unstable and only transiently formed and degrades again with reagent (F2), generating back reactant R together with waste (W2). The degradation reaction is catalysed by catalyst C2 with rate constant *k*_cat2_ and an uncatalysed background reaction with constant *k*_2_. We assume that the reactions are first order in catalyst C1 and C2 and not take into account any intermediate reaction steps, so only use the essential rate determining steps (*k*_cat_).

### Batch and CSTR non-equilibrium systems

**Fig. 1:**
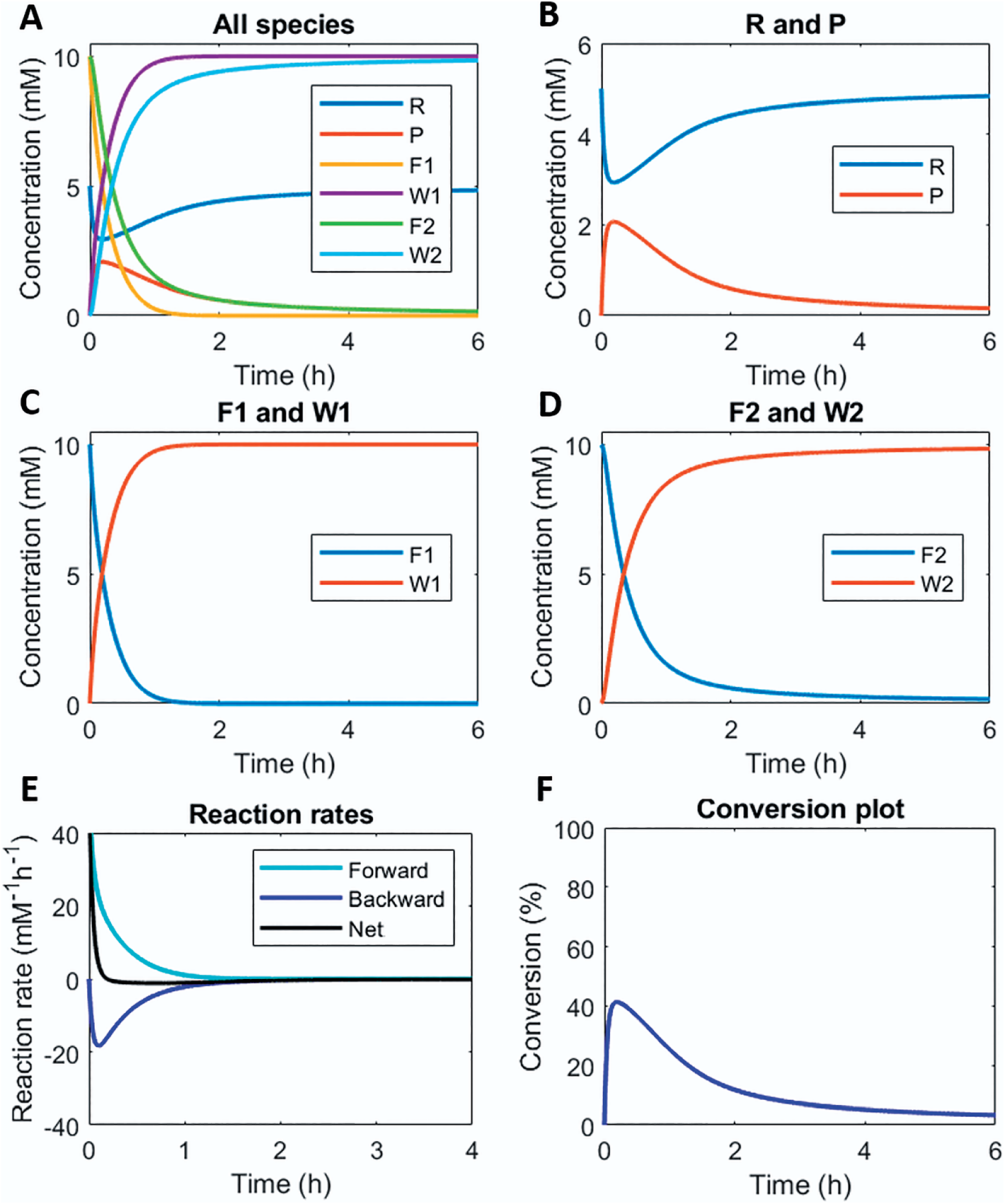
Numerical modelling output in batch-mode: (A) concentration of all species as a function of time. (B) Concentration of reactant R and product P as a function of time. (C) Concentration of fuel F1 and waste W1 as a function of time. (D) Concentration of reagent F2 and waste W2 as a function of time. (E) Reaction rates for product P formation and degradation as a function of time. (F) Conversion plot of reactant R. Initial conditions: [F1] = 10 mM, [W1] = 0 mM, [F2] = 10 mM, [W2] = 0 mM, [R] = 5 mM, [P] = 0 mM, [C1] = 0.1 mM, [C2] = 0.1 mM and rate constants *k*_cat1_ = 10 mM^−2^ h^−1^, *k*_1_ = 0.1 mM^−1^ h^−1^, *k*_cat2_ = 10 mM^−2^ h^−1^, *k*_2_ = 0.1 mM^−1^ h^−1^. N.B. in multiple out-of-equilibrium CRNs reagent F2 is the solvent (*e.g.* water). Then, the rate equation becomes independent of the F2 concentration.

Numerically solving this CRN in batch-mode gives rise to profiles of concentration over time of all species. Fig. 1A–D shows the concentration profiles as a function of time, when the concentrations of C1 and C2 are kept equal. In the batch-mode the fuel supply is finite and hence the non-equilibrium state is of temporary nature, resulting in a transient profile of product P and reactant R (Fig. 1B). Fuel (F1) and waste (W1) are quickly interconverted into each other (Fig. 1C), whereas reagent (F2) and waste (W2) interconversion is slightly delayed (Fig. 1D), because the backward reaction only kicks in from the moment product P is formed. Corresponding reaction rate and conversion plots are given in Fig. 1E and F. At the start of the fuel cycle the forward reaction dominates over the backward reaction resulting in the net formation of product P (Fig. 1E). The backward reaction reaches its maximum rate when the concentration of product P is at its maximum. But as product P is consumed again the backward reaction also quickly slows down.

**Fig. 2:**
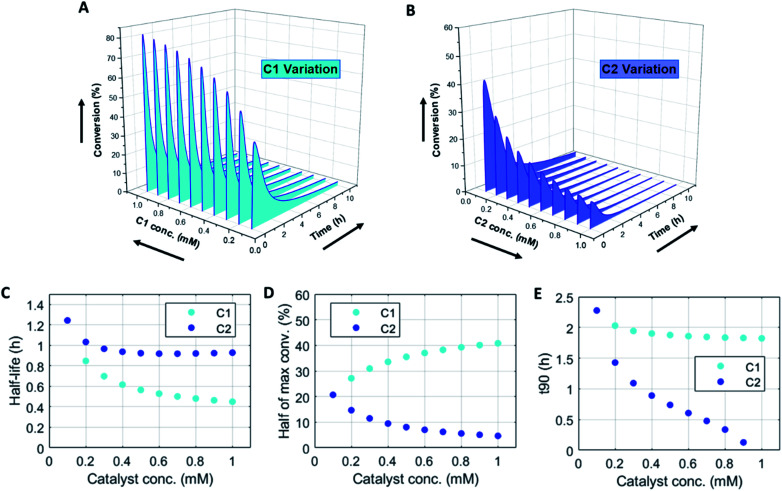
Numerical modelling output in batch-mode: (A) variation of catalyst C1 concentration from 0.1–1 mM. (B) Variation of catalyst C2 concentration from 0.1–1 mM. (C) Half-lives of the active state (P) for varying catalysts concentrations: C1 0.1–1 mM and C2 0.1–1 mM. (D) Half value of maximum conversion (after maximum) for varying catalysts concentrations: C1 0.1–1 mM and C2 0.1–1 mM. (E) Time (*t*90) to get back to 90% of reactant R (or 10% left of product P) for varying catalysts concentrations: C1 0.1–1 mM and C2 0.1–0.9 mM. N.B. the first data point in panels (C, D and E) coincide for C1 and C2. Conditions: [F1] = 10 mM, [W1] = 0 mM, [F2] = 10 mM, [W2] = 0 mM, [R] = 5 mM, [P] = 0 mM, [C1] = 0.1–1 mM, [C2] = 0.1–1 mM and rate constants *k*_cat1_ = 10 mM^−2^ h^−1^, *k*_1_ = 0.1 mM^−1^ h^−1^, *k*_cat2_ = 10 mM^−2^ h^−1^, *k*_2_ = 0.1 mM^−1^ h^−1^.

Changing the concentrations of C1 and C2 gives rise to maximization of conversions and lifetime of transient product P, as is apparent from Fig. 2A and B (for individual plots at different catalytic conditions see ESI Fig. S1–S6†). Increasing the C1 concentration leads to an increase in the maximum conversion of R, whereas the opposite holds true for C2 and a decrease in the maximum conversion is observed. Bringing one of the catalyst concentrations to zero results in prolonged lifetime of P (with no C2) (ESI Fig. S5†) or hardly any generation of P (with no C1) (ESI Fig. S6†). Furthermore, the half-lives of the active state P change upon catalyst variation (Fig. 2C and D). The half-life values (Fig. 2C) are defined as the time it takes when the maximum conversion is reduced by half. The corresponding values of half the maximum conversion are provided in Fig. 2D. Increasing concentrations of both C1 and C2 reduce the half-life of P (Fig. 2C). Without either one of the catalysts present the half-lives are much larger, 4.84 h (only C1) and 10.34 h (only C2) (data not shown in plot). Overall, addition of the catalysts increases the rates and with that the decay of product P. Interestingly, when we look at the *t*90 (time to recover 90% of reactant R) in Fig. 2E, changing the concentration of catalyst C2 seems to have more impact on the outcome of the *t*90. Increasing catalyst C2 from 0.1 to 0.9 mM brings the *t*90 almost to zero, while variations in C1 have little influence on the same *t*90 value. This observation is in contrast with the dependencies we found for the half-lives (Fig. 2C), where a changing C1 concentration gives a larger change in the half-life value. Noteworthy, when we switch off the uncatalysed background reactions (ESI Fig. S7†) the behaviour of the half-life and *t*90 for varying catalysts concentrations remains almost unaltered, suggesting this is a general phenomenon.

Several experimental systems with behaviour that is broadly described by this batch model, have been reported in the literature. This catalytic regulation to alter lifetimes and maximum conversions mostly been realized using enzyme catalysis. For instance, Ulijn and co-workers already in 2013 introduced a non-equilibrium CRN where α-chymotrypsin catalyses both the formation and degradation of a transient hydrogel.^16^ The backward reaction was very sensitive to the protease concentration, while the forward reaction showed little change upon changing the catalyst concentration. Only the maximum gelator conversion was slightly changed by using a lower catalyst concentration. Hence, using just one catalyst for both formation and degradation seems to come at the cost of limited control over individual reaction pathways. Next to proteases and esterases,^17^, (de)phosphorylating enzymes are popular catalysts in out-of-equilibrium systems.^15,18–22^ Using different enzymes for each path, for instance a kinase and phosphatase couple, increases the level of control. Yet, using enzymes as catalysts still faces major bottlenecks, such as limited long-term stability under operational conditions and inhibition by generated waste products. It is worth discussing that in contrast to the reaction systems described above with two catalysts and two significant background reactions, enzyme catalysed CRNs often have little to no background reaction and the enzymes are essential to make and break the transient product. In such a scenario, with near zero rate background reactions, the simulated scenario would still give similar concentration profiles for equal amounts of catalysts C1 and C2 (Fig. S8†). However, when also the level of catalyst C2 is brought to zero the backward reaction no longer takes place and the conversion of reactant R into product P is the final equilibrium state (Fig. S9†). In zero-rate background reaction scenarios, the catalysts have complete control over the behaviour of the system.

Compared to using biocatalysis, up to date only a few examples of metal catalysis, organocatalysis and acid/base catalysis have been reported.^23–25^ As an illustrative example, Das and co-workers introduced a catalytic histidine moiety in a self-supporting hydrogel, exhibiting fuel-driven transient stability and cooperative catalysis.^26^ The transient hydrogel was generated by esterification of the carboxylic end group of lipid functionalized histidine amphiphile precursors using a water-soluble carbodiimide as chemical fuel in combination with a *p*-nitrophenol nucleophile. A cooperative catalytic effect was realized by the proximity of the imidazole group of histidine, boosting the ester hydrolysis rate in the assembled state and consequently the disassembly of the gel state. Overall, this approach shows much resemblance with the enhanced GTPase activity of microtubules in living systems. The analogy to microtubules is also exemplified in follow-up works where the system shows designed negative feedback from the assembled structure, even harnessing temporal regulation of cross-β amyloid network electronic properties.^27,28^ Although this example from Das relies on organocatalysis, it is distinctly different from the simulation described here, because the catalyst (*i.e.* the histidine moiety) is integrated in the transient product. In that way, precise and predictable control over the yield of the transient product with the catalyst concentration is not straightforward, although it does show fascinating feedback control (*vide infra*).

Thus, with (two) catalysts participating in a non-equilibrium CRN it is obvious that a higher degree of control can be achieved over the maximum conversion and lifetime (*i.e.* half-lives and *t*90) than with the fuel alone. The catalytic regulation is of kinetic origin by alteration of the forward and backward reaction rates. Yet, in batch-mode when the fuel reservoir is exhausted the system reaches its equilibrium again and only the supply of new fuel can bring the system in a non-equilibrium state. Compared to these batch systems, supplying fuel in a continuous manner can give rise to a higher degree of control and for prolonged time.

**Fig. 3:**
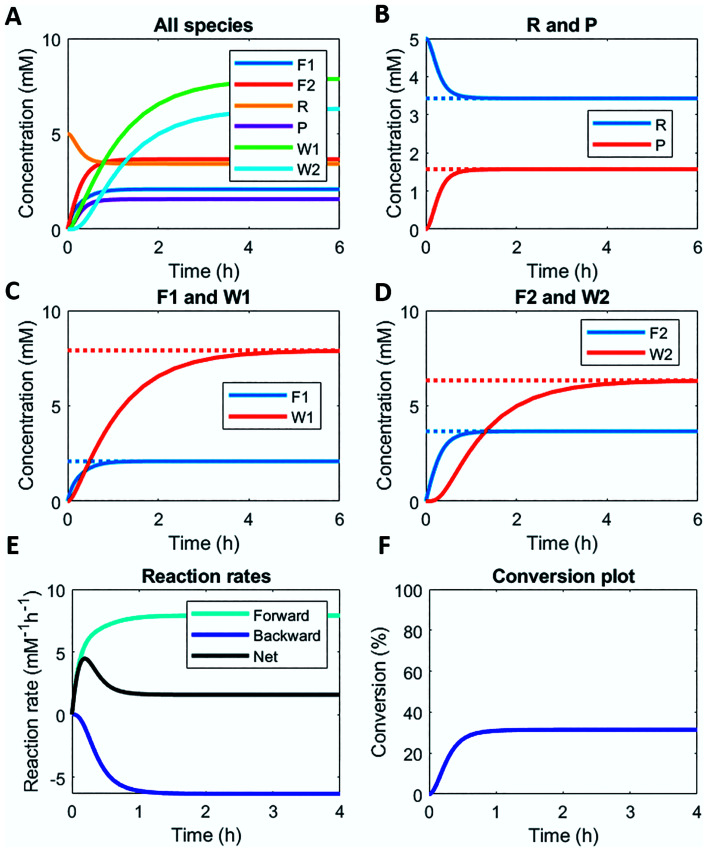
Numerical modelling output in CSTR: (A) concentration of all species as a function of time. (B) Concentration of reactant R and product P as a function of time (solid lines) and their steady states (dashed lines). (C) Concentration of fuel F1 and waste W1 as a function of time (solid lines) and their steady states (dashed lines). (D) Concentration of reagent F2 and waste W2 as a function of time (solid lines) and their steady states (dashed lines). (E) Reaction rates for product P formation and degradation as a function of time. (F) Conversion plot of reactant R. Inlet conditions: [F1] = 10 mM, [W1] = 0 mM, [F2] = 10 mM, [W2] = 0 mM, [R] = 5 mM, [P] = 0 mM, [C1] = 0.1 mM, [C2] = 0.1 mM, rate constants *k*_cat1_ = 10 mM^−2^ h^−1^, *k*_1_ = 0.1 mM^−1^ h^−1^, *k*_cat2_ = 10 mM^−2^ h^−1^, *k*_2_ = 0.1 mM^−1^ h^−1^ and residence time *τ* = 1 h.

Here, we demonstrate this by modelling the same reaction network (Scheme 1) in a continuous stirred tank reactor (CSTR). The catalyst concentrations can be varied to achieve different non-equilibrium steady states. Fig. 3 illustrates the concentration profiles of all species, when both catalysts are supplied in equal amounts. For product P and reactant R a non-equilibrium steady state is reached at around 1 h (Fig. 3B). Steady state profiles of both fuel and reagent (F1 and F2) are reached around 1 h as well, whereas the waste products (W1 and W2) reach their steady state some time later around 4 h (Fig. 3C and D). Because the fuel is supplied in a continuous fashion there is a continuous net formation of product P (Fig. 3E), which results in a continuous conversion around 30% (Fig. 3F).

Altering the catalyst ratios for this CSTR system changes the steady states levels of the various species. Supplying high amounts (1 mM and 0.1 mM) of either catalyst results in a fast establishment of the non-equilibrium steady states (ESI Fig. S10 and S11†), while lower catalyst concentrations (0.1 mM and 0.01 mM) extend the time to reach steady state (ESI Fig. S12 and S13†). Increasing [C1] relative to [C2] brings the steady state conversion above 40% (ESI Fig. S10† – C1 1 mM and C2 0.1 mM) or at 60% (ESI Fig. S12† – C1 0.1 mM and C2 0.01 mM). Lowering [C1] relative to [C2] brings the conversion around 12% (ESI Fig. S13† – C1 0.1 mM and C2 0.01 mM) or even below 10% (ESI Fig. S11† – C1 0.1 mM and C2 1 mM).

**Fig. 4:**
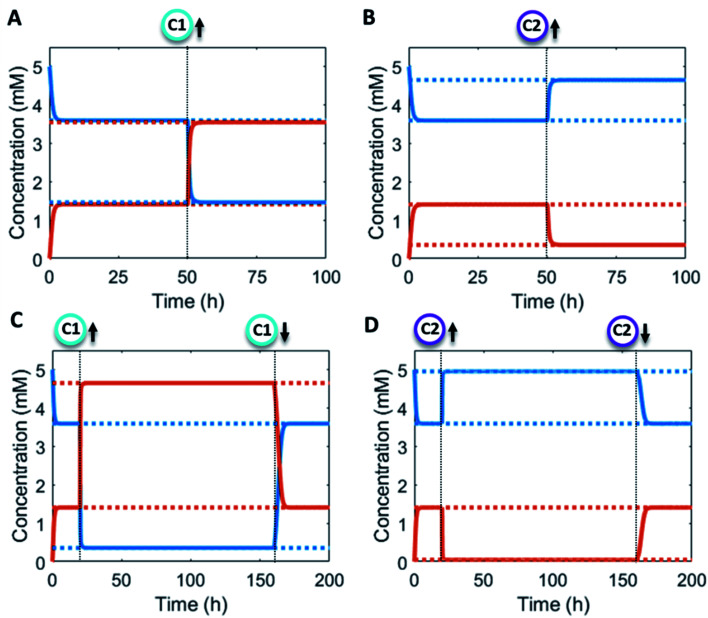
Numerical modelling output in CSTR system with *in situ* catalyst concentration change: (A) concentration of product P (red/orange line) and reactant R (blue line) as a function of time (solid lines) and their steady states (dashed lines). C1 = 0 mM, 0.1 mM (50 h) and C2 = 0 mM constant. (B) Concentration of product P (red/orange line) and reactant R (blue line) as a function of time (solid lines) and their steady states (dashed lines). C1 = 0 mM constant and C2 = 0 mM, 0.1 mM (50 h). (C) Concentration of product P (red/orange line) and reactant R (blue line) as a function of time (solid lines) and their steady states (dashed lines). C1 = 0 mM, 1 mM (20 h), 0 mM (160 h) and C2 = 0 mM constant. (D) Concentration of product P (red/orange line) and reactant R (blue line) as a function of time (solid lines) and their steady states (dashed lines). C1 = 0 mM constant and C2 = 0 mM, 1 mM (20 h), 0 mM (160 h). Inlet conditions: [F1] = 10 mM, [W1] = 0 mM, [F2] = 10 mM, [W2] = 0 mM, [R] = 5 mM, [P] = 0 mM, rate constants *k*_cat1_ = 10 mM^−2^ h^−1^, *k*_1_ = 0.1 mM^−2^ h^−1^, *k*_cat2_ = 10 mM^−2^ h^−1^, *k*_2_ = 0.1 mM^−1^ h^−1^ and residence time *τ* = 1 h.

Additionally, the catalyst concentrations can be altered *in situ* (Fig. 4). An increase in catalyst C1 results in an upregulation of the forward reaction and hence an increase in the steady state level of product P (red/orange line – Fig. 4A). Conversely, increasing C2 results in a downregulation of the net formation rate and a decrease in the steady state level of P (red/orange line – Fig. 4B). Over the course of the reaction up and downregulation of C1 or C2 can be performed sequentially to create a temporary up or downregulated steady state of P (Fig. 4C and D). Interestingly, the sequential up and downregulation resembles the onset of a chemical signal, which terminates again when the catalyst is removed from the reaction cycle. In this way, catalytic regulation can provide a high degree of control over the system.

In the literature, examples of continuous flow fuel-driven out-of-equilibrium chemical reaction networks are less common.^5^ Hermans and co-workers used an enzyme catalysed approach with a fuel-driven out-of-equilibrium systems based on ATP-fuelled phosphorylation to switch between right-handed and left-handed helix conformations of a peptide-perylenediimide supramolecular polymer.^15^ They could achieve various non-equilibrium steady states in a membrane reactor with a continuous flow set-up. Switching between steady states was also realized with regulation of the amount of fuel, but not with the catalysts, as fixed concentrations of enzymes were compartmentalized inside the reactor.

**Fig. 5:**
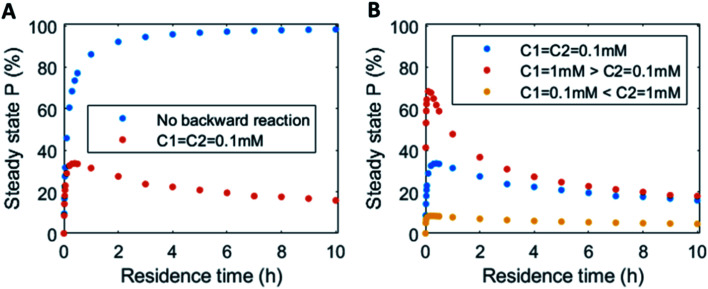
Residence time variation *vs.* the steady state concentration of product P: (A) uel cycle with equal catalysts concentrations (red symbols) *vs.* no backward reaction present (blue symbols). (B) Fuel cycle with varying catalysts concentrations: equal catalysts concentrations C1 = C2 = 0.1 mM (blue symbols), catalyst C1 1 mM > catalyst C2 0.1 mM (red symbols) and catalyst C2 1 mM > catalyst C1 0.1 mM (yellow symbols). Inlet conditions: [F1] = 10 mM, [W1] = 0 mM, [F2] = 10 mM, [W2] = 0 mM, [R] = 5 mM, [P] = 0 mM, [C1] = 0.1/1 mM, [C2] = 0.1/1 mM, rate constants *k*_cat1_ = 10 mM^−2^ h^−1^, *k*_1_ = 0.1 mM^−1^ h^−1^, *k*_cat2_ = 10 mM^−2^ h^−1^, *k*_2_ = 0.1 mM^−1^ h^−1^ and residence time variation from *τ* = 0–10 h. N.B. for no backward reactions, the rate of the catalysed and uncatalysed backward reactions were set to zero.

In continuous flow systems, in general not only the catalyst concentrations can be tuned, but also the residence time is an important design criterion. A shorter residence time decreases the time needed to reach the steady states (ESI Fig. S14†), while for longer residence times the steady states are reached later (ESI Fig. S15†). However, because of the non-equilibrium nature, there is an optimal residence time for reaching a maximum level of the product steady state. In Fig. 5 the steady state concentration of product P is plotted *versus* the residence time. For a system without backward reaction, increasing the residence time will keep increasing the steady state concentration (blue symbols – Fig. 5A). Yet, for a non-equilibrium system, where the product is also degraded at the same time, an optimum in residence time is observed (red/orange symbols – Fig. 5A). The height of this optimum can be altered accordingly by changing the catalyst ratios (Fig. 5B).

### Feedback regulation

Biasing of pathways in a CRN using two separate catalysts offers a high level of control under batch and flow conditions. Yet, inclusion of additional feedback elements can make the system even more adaptive. Feedback loops are a powerful strategy in cell regulation and used in, amongst others, gene transcription processes and maintenance of protein concentrations levels. A negative feedback speeds up the response to an activating input and steady state levels can be reached more quickly. This way, feedback elements are useful for the protection of cells from perturbations and make them robust towards environmental changes.^8^ Incorporation of feedback elements in synthetic materials can mimic cellular homeostasis and ultimately have a huge impact on biomedical designs, such as medical implants.^29–31^

Functionalities like oscillations and bifurcations, often found in non-equilibrium systems, are in essence composed of simpler network motifs combined with positive and negative feedback loops.^7,32^ A classic example of a bistable system and the basis of some oscillatory systems is an autocatalytic reaction in a CSTR.^33,34^ Due to the inherent non-linearity the system shows hysteresis in the reactant efflux *vs.* feed rate.^35^ Illustrative examples of oscillatory CRNs with integrated feedback elements come from the hands of Semenov, Huck and Whitesides.^5,36^ Specifically, Semenov and Whitesides designed a system based on thiol chemistry, whereas Semenov and Huck used enzyme activation and inhibition to arrive at a dynamic oscillatory system. Both systems feature an autocatalytic reaction (positive feedback) and an inhibition reaction (negative feedback) in a CSTR.

**Scheme 2:**
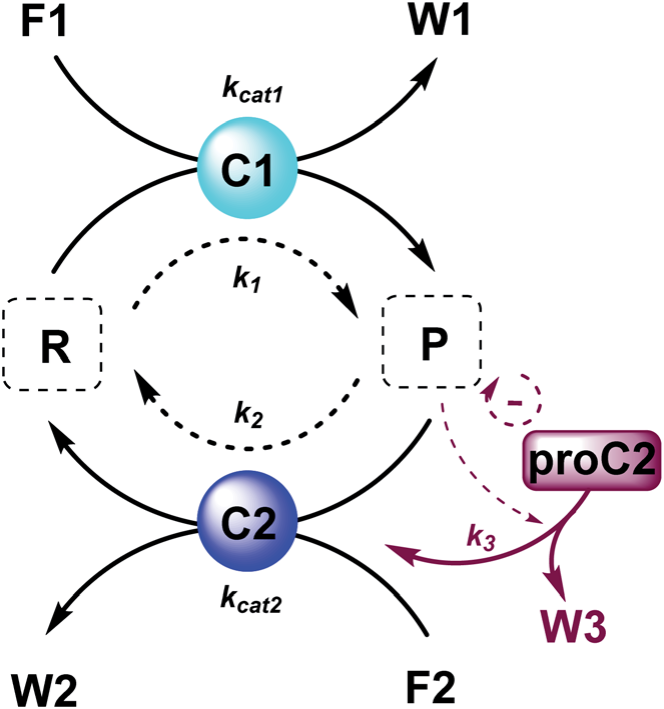
Generic fuel-driven CRN with (un)catalysed formation and degradation reactions and an additional procatalyst activation by product P. Product P catalyses the formation of catalyst C2 and waste W3 from procatalyst proC2. Product P is thus responsible for an acceleration of its own degradation.

**Fig. 6:**
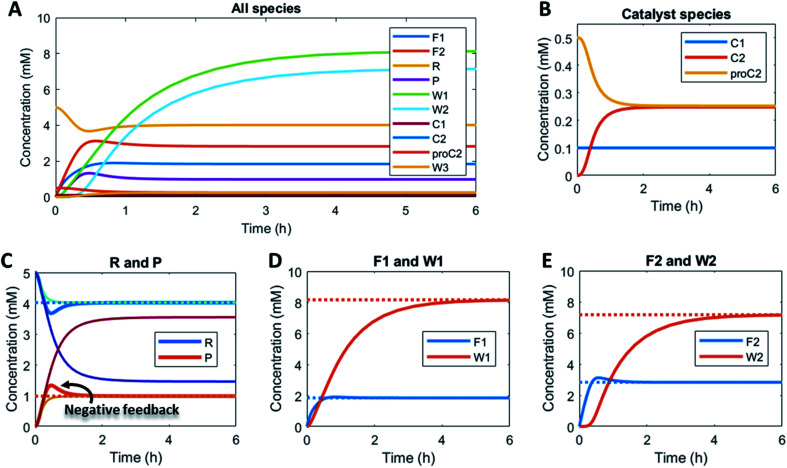
Numerical modelling output in CSTR with procatalyst activation by product P: (A) Concentration of all species as a function of time. (B) Concentration over time of the catalyst species: C1, C2 and proC2. (C) Concentration of reactant R and product P as a function of time (red and blue solid lines) and their steady states (red and blue dashed lines). The light blue and yellow line represent the scenario without feedback (without proC2, but 0.25 mM C2 from the start), while the dark blue line and dark red line give the outcome of the scenario without feedback and without C2 (0 mM from the start). (D) Concentration of fuel F1 and waste W1 as a function of time (solid lines) and their steady states (dashed lines). (E) Concentration of reagent F2 and waste W2 as a function of time (solid lines) and their steady states (dashed lines). Inlet conditions: [F1] = 10 mM, [W1] = 0 mM, [F2] = 10 mM, [W2] = 0 mM, [R] = 5 mM, [P] = 0 mM, [C1] = 0.1 mM, [C2] = 0 mM, [proC2] = 0.5 mM, [W3] = 0 mM rate constants *k*_cat1_ = 10 mM^−2^ h^−1^, *k*_1_ = 0.1 mM^−1^ h^−1^, *k*_cat2_ = 10 mM^−2^ h^−1^, *k*_2_ = 0.1 mM^−1^ h^−1^, *k*_3_ = 1 mM^−1^ h^−1^ and residence time *τ* = 1 h.

Here, to illustrate how a simple feedback loop can be incorporated in this generic fuel-driven CRN, we add an additional reaction step (Scheme 2 – reaction in maroon colour). Instead of having both catalysts C1 and C2 from the beginning in the reaction mixture, we introduce an inactive procatalyst^37–39^ for C2, which can be converted into the active catalyst C2 and a waste product (W3). This activation is catalysed by product P itself. In this way, product P is responsible for an acceleration of its own degradation, which creates an extra negative feedback element. In Fig. 6 the concentration profiles over time are given for all the species (A), the catalyst species (B), reactant R and product P (C), fuel F1 and waste W1 (D) and reagent F2 and waste W2 (E). Catalyst C2 is generated from proC2 (Fig. 6B) and both species reach a steady state around 2 h. Because of the increase in C2 over time, product P (and reactant R), show(s) a bump in the concentration profile. At first the concentration of P increases rapidly because its degradation is limited due to a low C2 concentration. However, as more P is generated also more C2 is generated and hence the concentration of P starts to drop again, after which it reaches a steady state. As such, a simple catalytic activation step can change the behaviour of the system significantly and introduce a catalytic negative feedback of the product on its own production.

## Perspective and outlook

Catalysis is extremely important in biological processes and regulates many non-equilibrium biological CRNs. In these natural systems, catalysis is performed by enzymes. Recently, examples of man-made fuel-driven non-equilibrium CRNs have been growing in number. Incorporation of catalysis in such systems is not trivial, especially because of the high degree of complexity including many simultaneous chemical reactions and reactive chemical functionalities. Nonetheless, several examples of artificial non-equilibrium systems with enzyme, transition metal, organocatalysis and acid catalysis have been developed in recent years.^15–21,23–26,40,41^ The majority of these examples exploit enzyme catalysis to regulate maximum conversion and lifetime. Only a small number of transition metal and organocatalytically regulated out-of-equilibrium systems is reported to date. Expanding nature's toolbox by using artificial catalysts, such as transition metals, simpler small molecule organocatalysts or even acid/base catalysis could significantly boost the application potential of this field. Especially organocatalysts can be easily modified and applied in soft materials or bulk polymer materials,^42,43^ although they do often suffer from low activity and high background reactivity. Transition metal catalysts generally outperform organocatalysts when it comes to activity and on/off-ratios. Incorporation of catalysis in materials could open up a new type of matter with a more dynamic character. As a proof of principle, we showed here, using a kinetic model, how two general catalysts can influence the behavior of a fuel-driven non-equilibrium CRN. Altering catalysts' levels in a batch system results in varying maximum conversion and lifetime (expressed in half-life and *t*90 values, where both parameters respond differently to changes in either catalyst concentration). Alternatively, the same system in a CSTR can switch between different non-equilibrium steady states depending on the catalysts' ratios. *In situ* up and downregulation of catalysts' concentrations gives a temporary rise in the product steady state. Additionally, the system can be designed with a negative feedback element, altering the concentration over time profile.

Having demonstrated that catalytic regulation in a fuel-driven non-equilibrium CRN provides a high degree of control in the simulated system, the next step would be to find chemical reactivity, especially catalysts, that can be used for the design of catalytic non-equilibrium CRNs. Moreover, coupling catalysis to a dissipative CRN containing assembling products could give rise to unusual assembly behaviour and feedback.^26–28,44^ Here, instead of using enzymes, with limited operational stability and high specificity, it would be recommended to start exploring small molecules and metal catalysts. This way organocatalysis in combination with metal catalysis in non-natural systems can play similar roles as enzymes in nature and provide new systems with a high degree of control and an adaptive character. Ultimately, a better understanding of such systems and the associated chemistry can bring us closer to the design of man-made signal responsive and interactive materials.^29–31,45^

## Author contributions

M. P. v. d. H. and T. d. B. developed the kinetic models and analyzed the data. M. P. v. d. H. and R. E. conceived the overall research. M. P. v. d. H. wrote the manuscript. R. E. revised the manuscript and directed the research. All authors commented on the work and the manuscript.

## Conflicts of interest

The authors declare no competing financial interest.

## Supplementary Material

SC-012-D0SC06406H-s001

SC-012-D0SC06406H-s002

SC-012-D0SC06406H-s003
