# Supplementary material for: On the use of catalysis to bias reaction pathways in out-of-equilibrium systems
Source: Chem Sci. 2021 Feb 9;12(12):4484–93. doi: 10.1039/d0sc06406h (PMC8179475; doi:10.1039/d0sc06406h)
Supplement: SC-012-D0SC06406H-s003 [file SC-012-D0SC06406H-s003.pdf]

Electronic Supplementary Material (ESI) for Chemical Science.
This journal is © The Royal Society of Chemistry 2021

Supporting information

On the use of catalysis to bias reaction pathways in out-of-equilibrium systems

Michelle P. van der Helm,[1] Tuanke de Beun,[1] and Rienk Eelkema*,[1]

[1] Department of Chemical Engineering, Delft University of Technology,
Van der Maasweg 9, 2629 HZ Delft, The Netherlands

***Corresponding Author.**

Tel: +31 (0)15 27 81035; E-mail: R.Eelkema@tudelft.nl.

1 Kinetic model design

The fuel-driven CRN consists of four reactions, two catalysed formation and degradation reactions and two uncatalysed formation and degradation reactions. A numerical model for this reaction network was written in MATLAB R2018b. Reactant (R) is interconverted into product (P) by suppling fuel (F1) and concomitantly generating waste (W1), catalysed by C1 ($kcat_1$) and uncatalysed (k_1). P is only transiently stable and degrades again with reagent (F2), generating R and waste (W2), catalysed by C2 ($kcat_2$) and uncatalysed (k_2). This results in the following system of differential equations (ODEs):

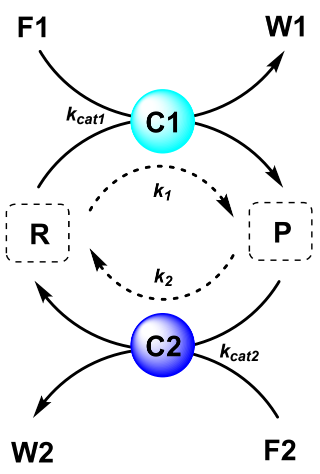

$$\frac{d[P]}{dt} = (k_{cat1}[C1] + k_1)[F1][R] - (k_{cat2}[C2] + k_2)[F2][P]$$

$$\frac{d[R]}{dt} = -(k_{cat1}[C1] + k_1)[F1][R] + (k_{cat2}[C2] + k_2)[F2][P]$$

$$\frac{d[F1]}{dt} = -(k_{cat1}[C1] + k_1)[F1][R]$$

$$\frac{d[F2]}{dt} = -(k_{cat2}[C2] + k_2)[F2][P]$$

$$\frac{d[W1]}{dt} = (k_{cat1}[C1] + k_1)[F1][R]$$

$$\frac{d[W2]}{dt} = (k_{cat2}[C2] + k_2)[F2][P]$$

The system of ODEs is solved numerically in batch and continuous flow (i.e. CSTR) for different initial/inlet conditions, which are all specified in the caption of each figure in the main text and the supporting info.

For the CSTR system the inflow and outflow of reactants and products is taken into account, following the equation for all species:

$$Accumulation = in - out + reaction$$

$$\frac{d[C]}{dt} = \frac{C_{in} - C_{out}}{\tau} + reaction$$

In order to calculate the steady states levels in the CSTR system the differential equations are set to zero and calculated numerically with MATLAB:

In steady state

$$\frac{d[C]}{dt} = \frac{C_{in} - C_{out}}{\tau} + reaction = 0$$

For the system with an additional negative feedback element, the procatalyst activation step catalysed by product P leads to the introduction of the following extra rate equations to the system of ODEs:

System with procatalyst activation for C2 (catalysed by product P)

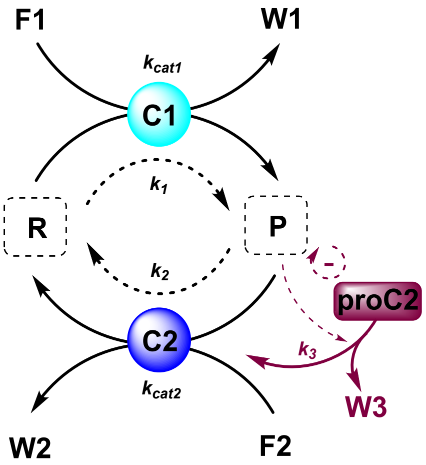

$$\frac{d[proC2]}{dt} = -k_3[P][proC2]$$

$$\frac{d[C2]}{dt} = k_3[P][proC2]$$

$$\frac{d[W3]}{dt} = k_3[P][proC2]$$

2 Batch reaction output

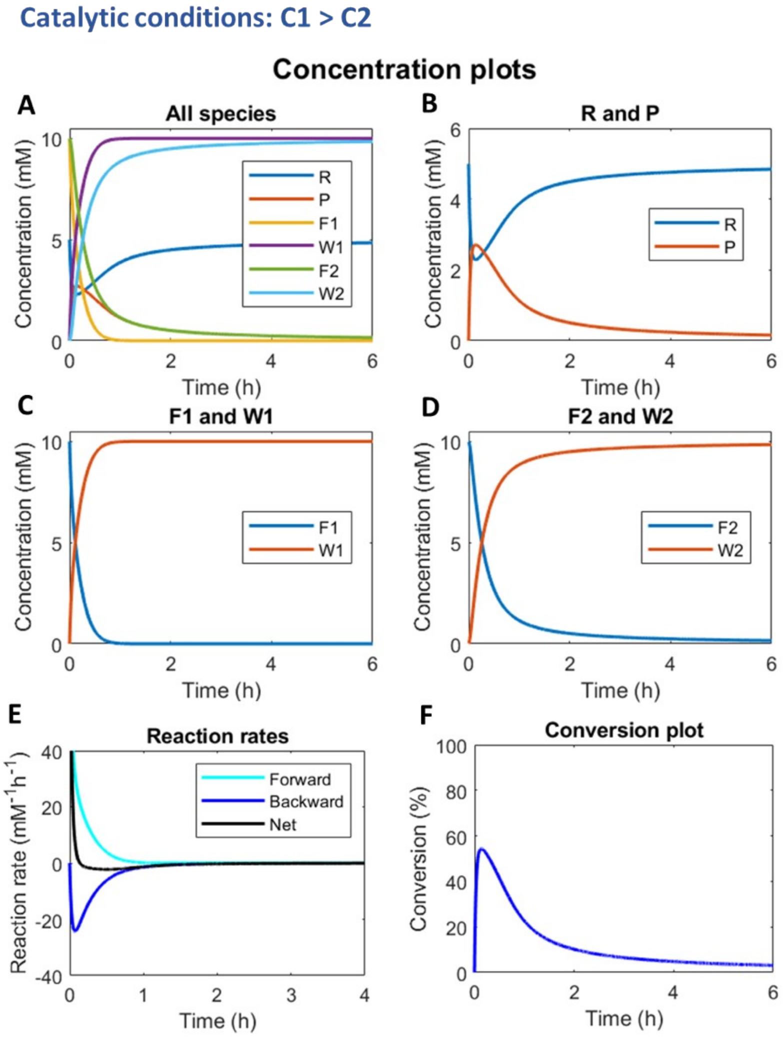

Figure S1: Numerical modelling output in batch-mode: **(A).** Concentration of all species as a function of time. **(B).** Concentration of reactant R and product P as a function of time. **(C).** Concentration of fuel F1 and waste W1 as a function of time. **(D).** Concentration of reagent F2 and waste W2 as a function of time. **(E).** Reaction rates for product P formation and degradation as a function of time. **(F).** Conversion plot of reactant R. Initial conditions: [F1]=10 mM, [W1]=0 mM, [F2]=10 mM, [W2]=0 mM, [R]=5 mM, [P]=0 mM, **[C1]=0.2 mM**, **[C2]=0.1 mM** and rate constants k_{cat1}= 10 $mM^{-2}h^{-1}$, k_1= 0.1 $mM^{-1}h^{-1}$, k_{cat2}= 10 $mM^{-2}h^{-1}$, k_2= 0.1 $mM^{-1}h^{-1}$.

Catalytic conditions: C1 < C2

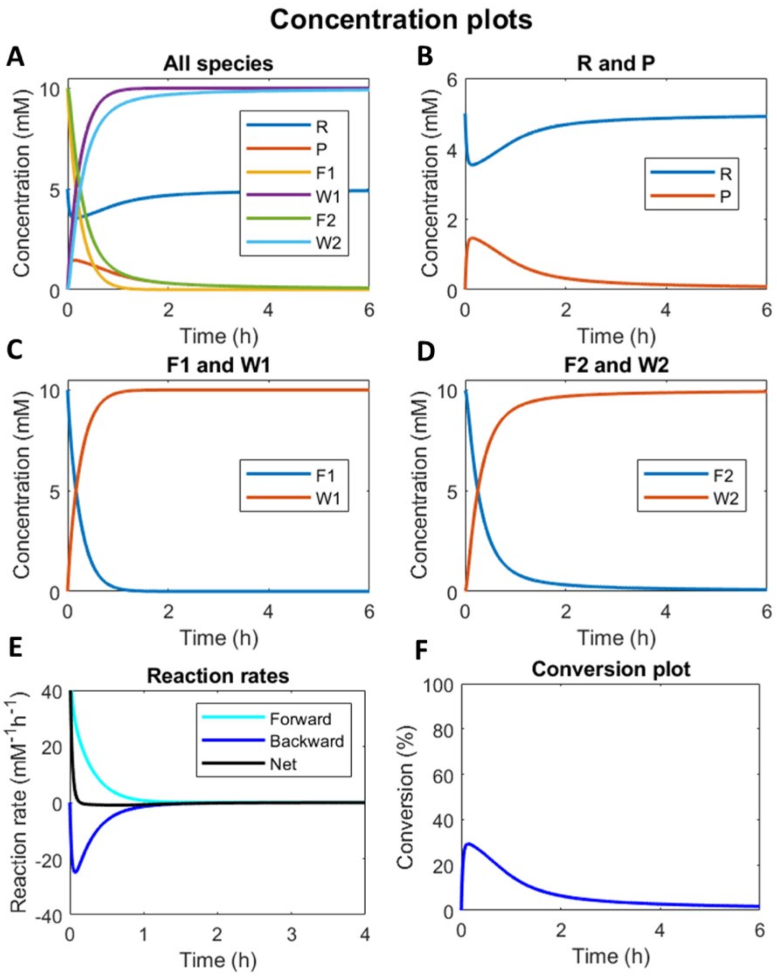

Figure S2: Numerical modelling output in batch-mode: **(A).** Concentration of all species as a function of time. **(B).** Concentration of reactant R and product P as a function of time. **(C).** Concentration of fuel F1 and waste W1 as a function of time. **(D).** Concentration of reagent F2 and waste W2 as a function of time. **(E).** Reaction rates for product P formation and degradation as a function of time. **(F).** Conversion plot of reactant R. Initial conditions: [F1]=10 mM, [W1]=0 mM, [F2]=10 mM, [W2]=0 mM, [R]=5 mM, [P]=0 mM, **[C1]=0.1 mM**, **[C2]=0.2 mM** and rate constants k_{cat1}= 10 $mM^{-2}h^{-1}$, k_1= 0.1 $mM^{-1}h^{-1}$, k_{cat2}= 10 $mM^{-2}h^{-1}$, k_2= 0.1 $mM^{-1}h^{-1}$.

Catalytic conditions: C1 >> C2

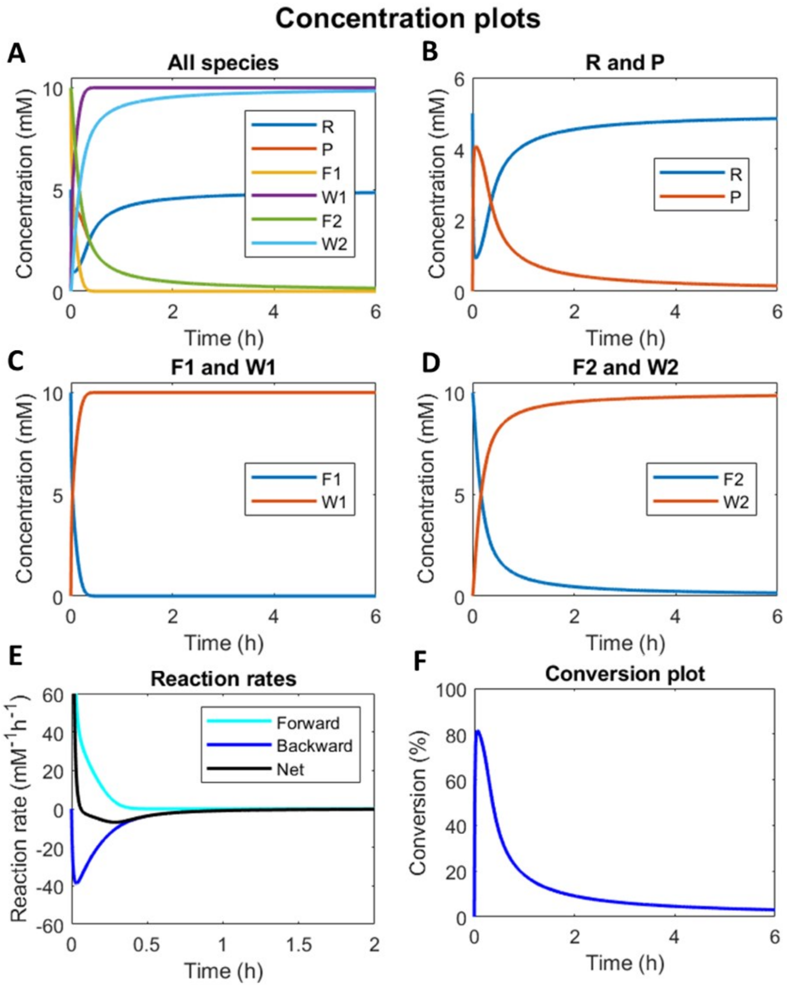

Figure S3: Numerical modelling output in batch-mode: **(A).** Concentration of all species as a function of time. **(B).** Concentration of reactant R and product P as a function of time. **(C).** Concentration of fuel F1 and waste W1 as a function of time. **(D).** Concentration of reagent F2 and waste W2 as a function of time. **(E).** Reaction rates for product P formation and degradation as a function of time. **(F).** Conversion plot of reactant R. Initial conditions: [F1]=10 mM, [W1]=0 mM, [F2]=10 mM, [W2]=0 mM, [R]=5 mM, [P]=0 mM, **[C1]=1 mM**, **[C2]=0.1 mM** and rate constants k_{cat1}= 10 $mM^{-2}h^{-1}$, k_1= 0.1 $mM^{-1}h^{-1}$, k_{cat2}= 10 $mM^{-2}h^{-1}$, k_2= 0.1 $mM^{-1}h^{-1}$.

Catalytic conditions: C1 << C2

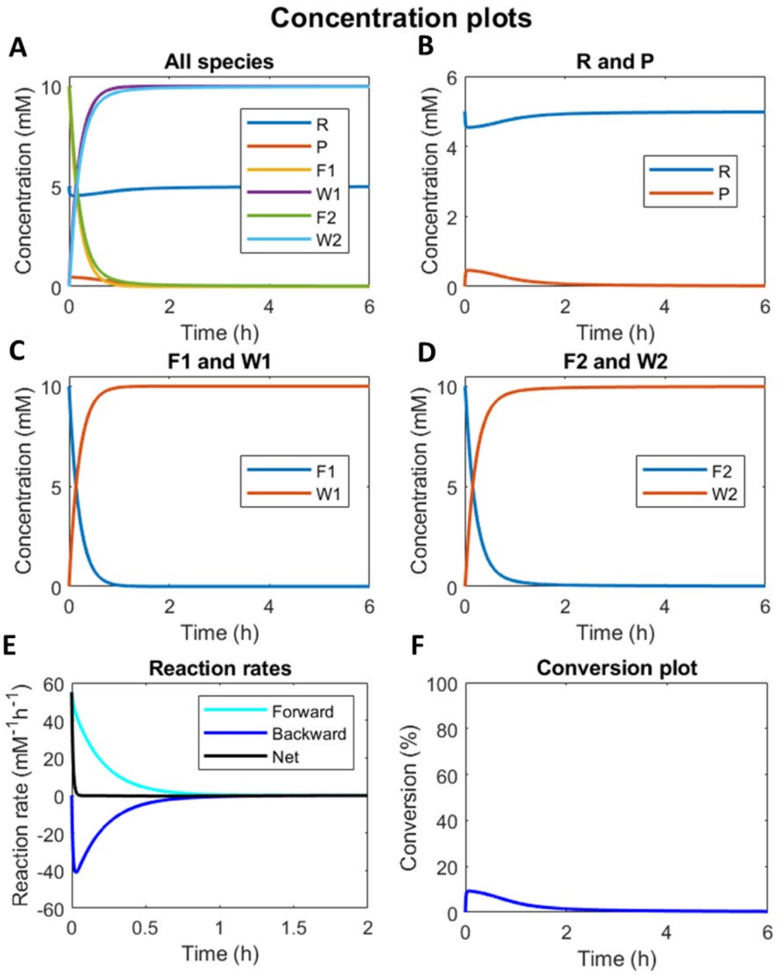

Figure S4: Numerical modelling output in batch-mode: **(A).** Concentration of all species as a function of time. **(B).** Concentration of reactant R and product P as a function of time. **(C).** Concentration of fuel F1 and waste W1 as a function of time. **(D).** Concentration of reagent F2 and waste W2 as a function of time. **(E).** Reaction rates for product P formation and degradation as a function of time. **(F).** Conversion plot of reactant R. Initial conditions: [F1]=10 mM, [W1]=0 mM, [F2]=10 mM, [W2]=0 mM, [R]=5 mM, [P]=0 mM, **[C1]=0.1 mM**, **[C2]=1 mM** and rate constants k_{cat1}= 10 mM^{-2}h^{-1}, k_1= 0.1 mM^{-1}h^{-1}, k_{cat2}= 10 mM^{-2}h^{-1}, k_2= 0.1 mM^{-1}h^{-1}.

Catalytic conditions: No C2

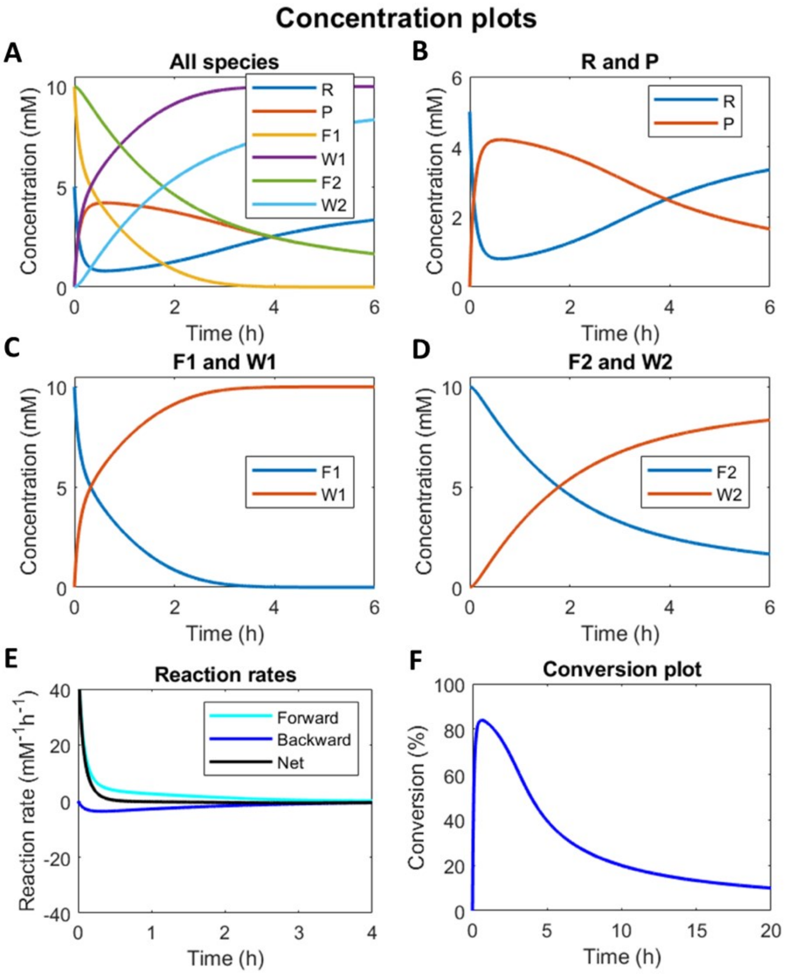

Figure S5: Numerical modelling output in batch-mode: **(A).** Concentration of all species as a function of time. **(B).** Concentration of reactant R and product P as a function of time. **(C).** Concentration of fuel F1 and waste W1 as a function of time. **(D).** Concentration of reagent F2 and waste W2 as a function of time. **(E).** Reaction rates for product P formation and degradation as a function of time. **(F).** Conversion plot of reactant R. Initial conditions: [F1]=10 mM, [W1]=0 mM, [F2]=10 mM, [W2]=0 mM, [R]=5 mM, [P]=0 mM, **[C1]=0.1 mM**, **[C2]=0 mM** and rate constants k_{cat1}= 10 $mM^{-2}h^{-1}$, k_1= 0.1 $mM^{-1}h^{-1}$, k_{cat2}= 10 $mM^{-2}h^{-1}$, k_2= 0.1 $mM^{-1}h^{-1}$.

Catalytic conditions: No C1

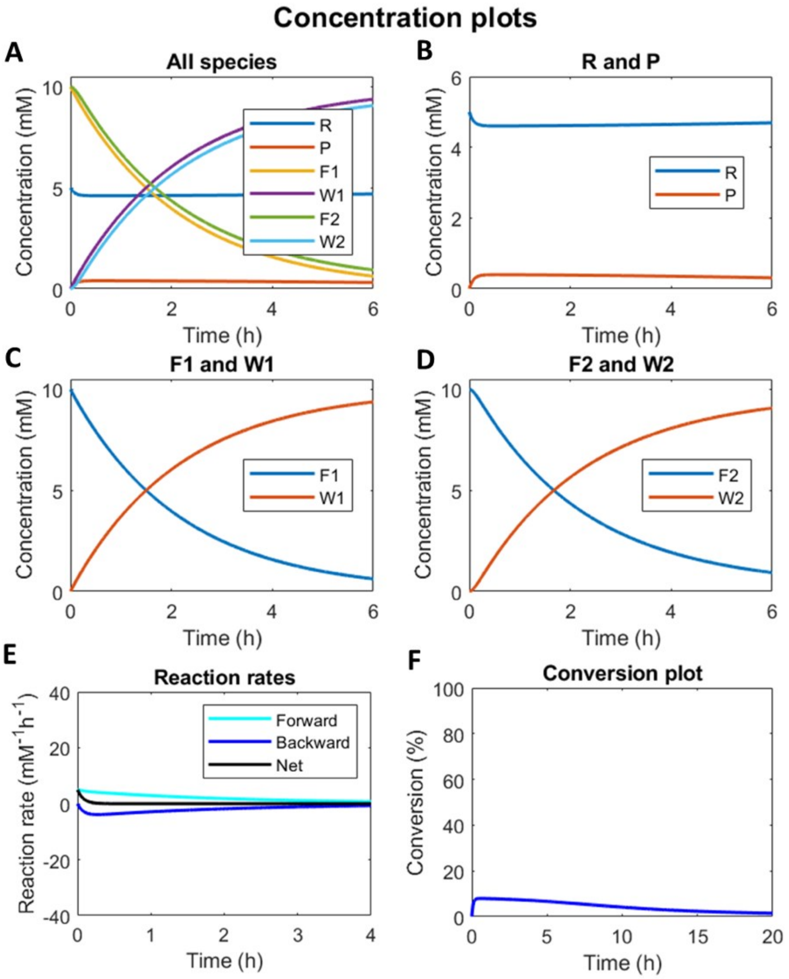

Figure S6: Numerical modelling output in batch-mode: **(A).** Concentration of all species as a function of time. **(B).** Concentration of reactant R and product P as a function of time. **(C).** Concentration of fuel F1 and waste W1 as a function of time. **(D).** Concentration of reagent F2 and waste W2 as a function of time. **(E).** Reaction rates for product P formation and degradation as a function of time. **(F).** Conversion plot of reactant R. Initial conditions: [F1]=10 mM, [W1]=0 mM, [F2]=10 mM, [W2]=0 mM, [R]=5 mM, [P]=0 mM, **[C1]=0 mM**, **[C2]=0.1 mM** and rate constants k_{cat1}= 10 $mM^{-2}h^{-1}$, k_1= 0.1 $mM^{-1}h^{-1}$, k_{cat2}= 10 $mM^{-2}h^{-1}$, k_2= 0.1 $mM^{-1}h^{-1}$.

Half-life and t90 for varying catalysts concentrations without background

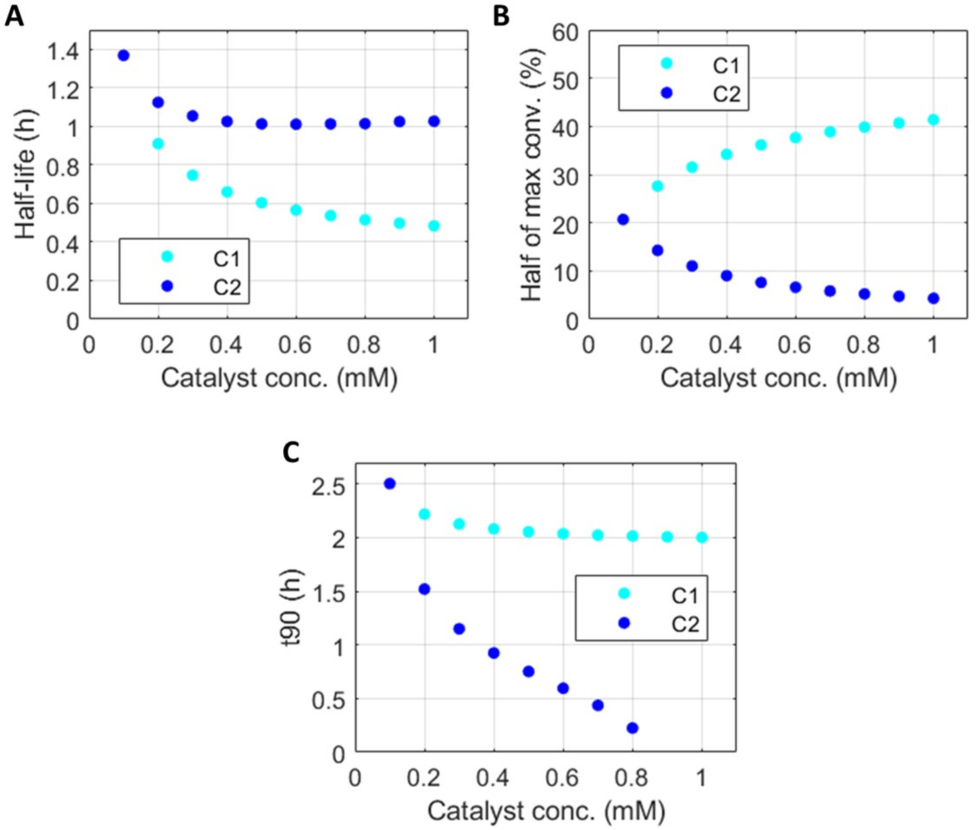

Figure S7: Numerical modelling output in batch-mode: **(A)** Half-lives of the active state (P) for varying catalysts concentrations: C1 0.1-1 mM and C2 0.1-1 mM. **(B)** Half value of maximum conversion (after maximum) for varying catalysts concentrations: C1 0.1-1 mM and C2 0.1-1 mM. **(C)** Time (t90) to get back to 90% of reactant R (or 10% left of product P) for varying catalysts concentrations: C1 0.1-1 mM and C2 0.1-0.8 mM. N.B. the first data point in panels (A, B and C) coincide for C1 and C2. Conditions: [F1]=10 mM, [W1]=0 mM, [F2]=10 mM, [W2]=0 mM, [R]=5 mM, [P]=0 mM, [C1]=0.1-1 mM, [C2]=0.1-1 mM and rate constants k_{cat1}=10 mM^{-2}h^{-1}, k_1=0 mM^{-1}h^{-1}, k_{cat2}=10 mM^{-2}h^{-1}, k_2=0 mM^{-1}h^{-1}.

Catalytic conditions: C1=C2, no background for forward and backward reaction

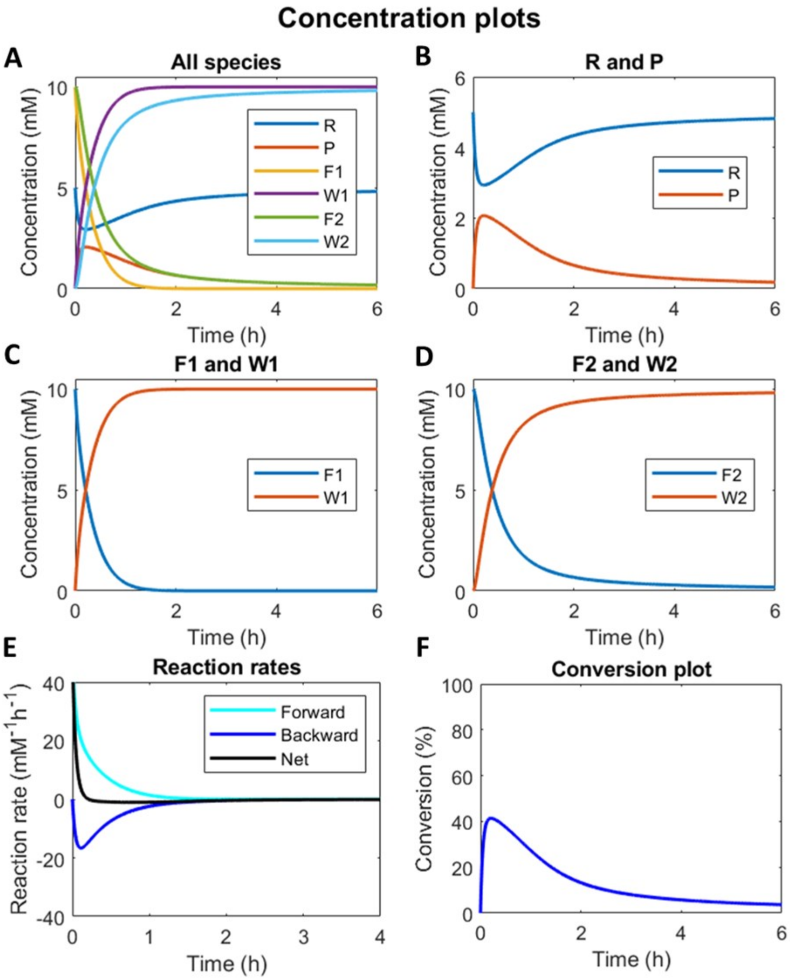

Figure S8: Numerical modelling output in batch-mode: **(A).** Concentration of all species as a function of time. **(B).** Concentration of reactant R and product P as a function of time. **(C).** Concentration of fuel F1 and waste W1 as a function of time. **(D).** Concentration of reagent F2 and waste W2 as a function of time. **(E).** Reaction rates for product P formation and degradation as a function of time. **(F).** Conversion plot of reactant R. Initial conditions: [F1]=10 mM, [W1]=0 mM, [F2]=10 mM, [W2]=0 mM, [R]=5 mM, [P]=0 mM, **[C1]=0.1 mM**, **[C2]=0.1 mM** and rate constants k_{cat1}= 10 $mM^{-2}h^{-1}$, $\boldsymbol{k_1}$**= 0** $\boldsymbol{mM^{-1}h^{-1}}$, k_{cat2}= 10 $mM^{-2}h^{-1}$, $\boldsymbol{k_2}$**= 0** $\boldsymbol{mM^{-1}h^{-1}}$.

Catalytic conditions: No C2 and no background for forward and backward reaction

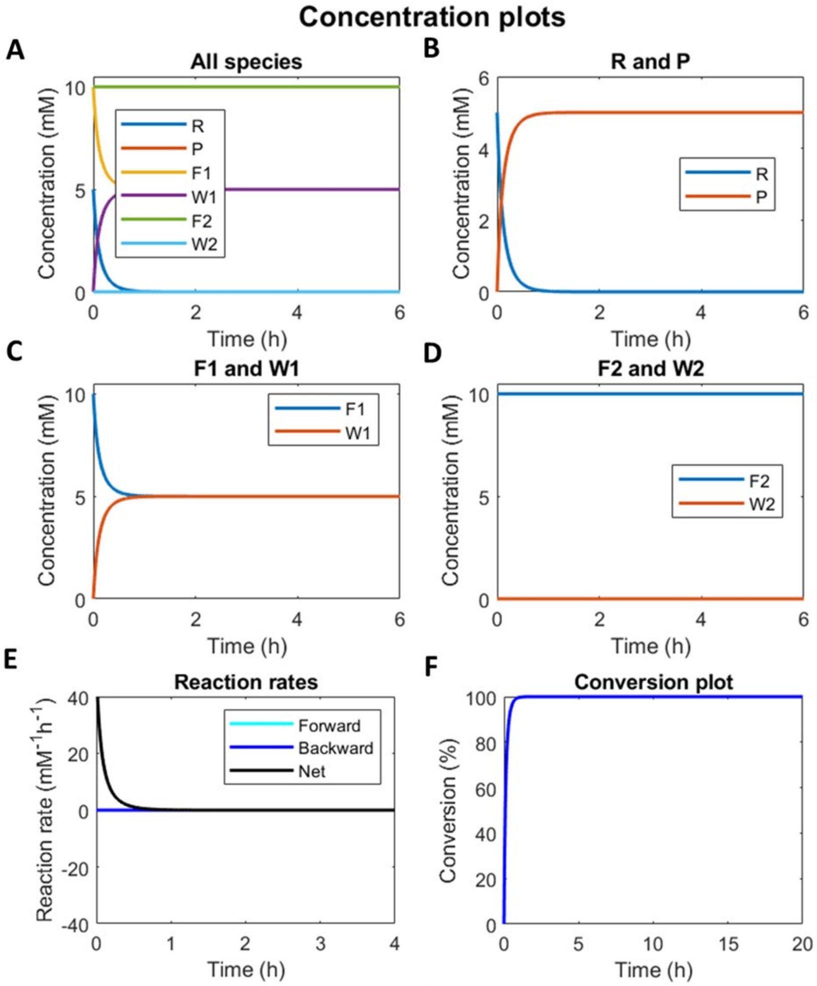

Figure S9: Numerical modelling output in batch-mode: **(A).** Concentration of all species as a function of time. **(B).** Concentration of reactant R and product P as a function of time. **(C).** Concentration of fuel F1 and waste W1 as a function of time. **(D).** Concentration of reagent F2 and waste W2 as a function of time. **(E).** Reaction rates for product P formation and degradation as a function of time. **(F).** Conversion plot of reactant R. Initial conditions: [F1]=10 mM, [W1]=0 mM, [F2]=10 mM, [W2]=0 mM, [R]=5 mM, [P]=0 mM, **[C1]=0.1 mM**, **[C2]=0 mM** and rate constants k_{cat1}= 10 $mM^{-2}h^{-1}$, $\boldsymbol{k_1}$**= 0** $\mathbf{mM^{-1}h^{-1}}$, k_{cat2}= 10 $mM^{-2}h^{-1}$, $\boldsymbol{k_2}$**= 0** $\mathbf{mM^{-1}h^{-1}}$.

3 CSTR reaction output

Catalytic conditions: C1 > C2

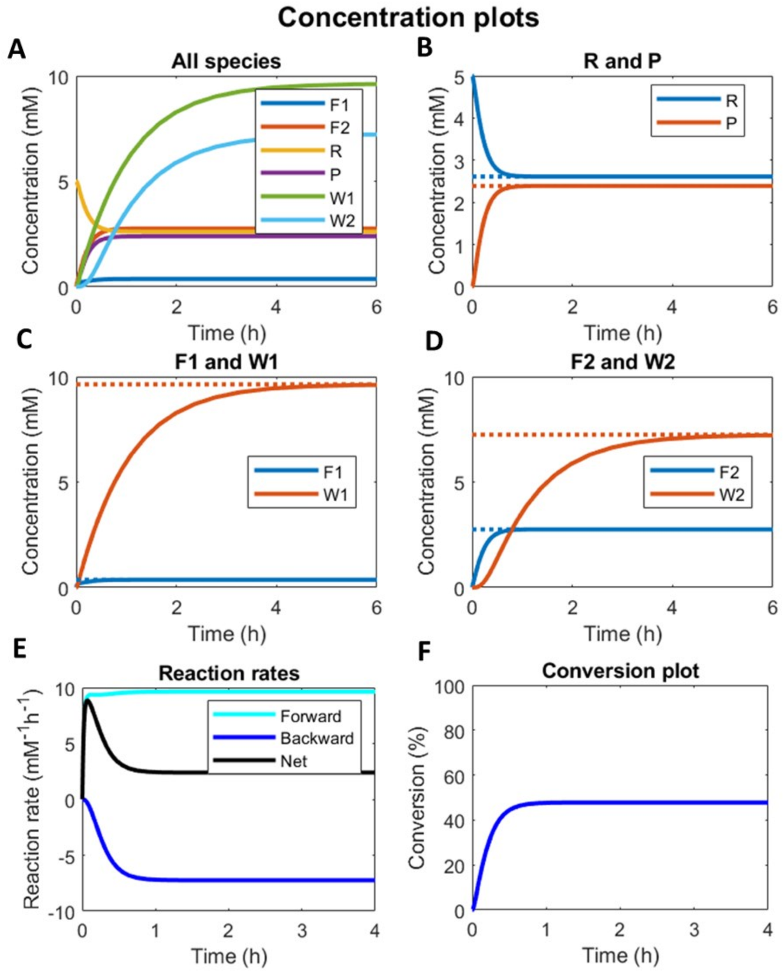

Figure S10: Numerical modelling output in CSTR: **(A).** Concentration of all species as a function of time. **(B).** Concentration of reactant R and product P as a function of time (solid lines) and their steady states (dashed lines). **(C).** Concentration of fuel F1 and waste W1 as a function of time (solid lines) and their steady states (dashed lines). **(D).** Concentration of reagent F2 and waste W2 as a function of time (solid lines) and their steady states (dashed lines). **(E).** Reaction rates for product P formation and degradation as a function of time. **(F).** Conversion plot of reactant R. Inlet conditions: [F1]=10 mM, [W1]=0 mM, [F2]=10 mM, [W2]=0 mM, [R]=5 mM, [P]=0 mM, **[C1]=1 mM, [C2]=0.1 mM**, rate constants k_{cat1}= 10 mM^{-2}h^{-1}, k_1= 0.1 mM^{-1}h^{-1}, k_{cat2}= 10 mM^{-2}h^{-1}, k_2= 0.1 mM^{-1}h^{-1} and **residence time τ = 1 h**.

Catalytic conditions: C1 < C2

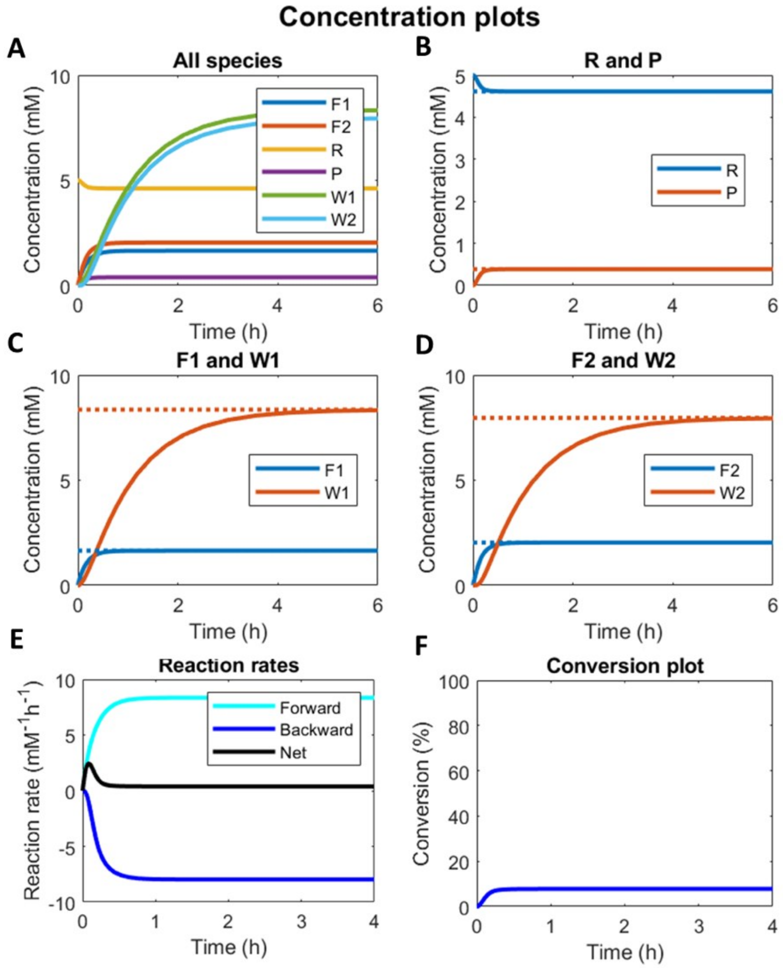

Figure S11: Numerical modelling output in CSTR: **(A).** Concentration of all species as a function of time. **(B).** Concentration of reactant R and product P as a function of time (solid lines) and their steady states (dashed lines). **(C).** Concentration of fuel F1 and waste W1 as a function of time (solid lines) and their steady states (dashed lines). **(D).** Concentration of reagent F2 and waste W2 as a function of time (solid lines) and their steady states (dashed lines). **(E).** Reaction rates for product P formation and degradation as a function of time. **(F).** Conversion plot of reactant R. Inlet conditions: [F1]=10 mM, [W1]=0 mM, [F2]=10 mM, [W2]=0 mM, [R]=5 mM, [P]=0 mM, **[C1]=0.1 mM, [C2]=1 mM**, rate constants k_{cat1}= 10 mM^{-2}h^{-1}, k_1= 0.1 mM^{-1}h^{-1}, k_{cat2}= 10 mM^{-2}h^{-1}, k_2= 0.1 mM^{-1}h^{-1} and **residence time τ = 1 h**.

Catalytic conditions: C1 > C2

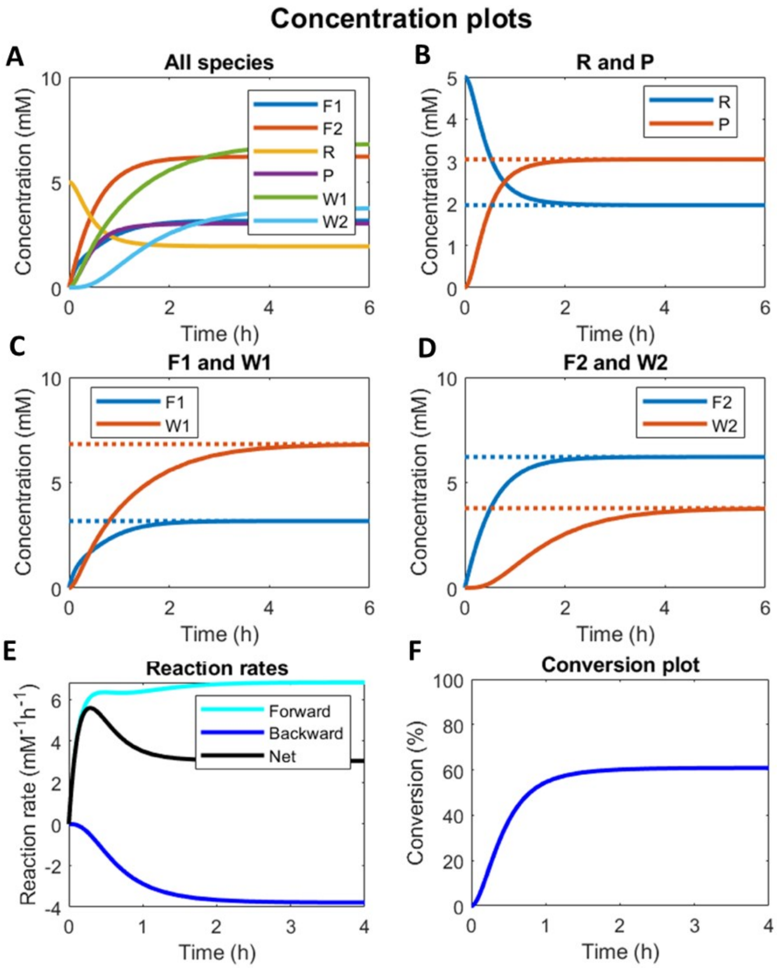

Figure S12: Numerical modelling output in CSTR: **(A).** Concentration of all species as a function of time. **(B).** Concentration of reactant R and product P as a function of time (solid lines) and their steady states (dashed lines). **(C).** Concentration of fuel F1 and waste W1 as a function of time (solid lines) and their steady states (dashed lines). **(D).** Concentration of reagent F2 and waste W2 as a function of time (solid lines) and their steady states (dashed lines). **(E).** Reaction rates for product P formation and degradation as a function of time. **(F).** Conversion plot of reactant R. Inlet conditions: [F1]=10 mM, [W1]=0 mM, [F2]=10 mM, [W2]=0 mM, [R]=5 mM, [P]=0 mM, **[C1]=0.1 mM, [C2]=0.01 mM**, rate constants k_{cat1}= 10 mM^{-2}h^{-1}, k_1= 0.1 mM^{-1}h^{-1}, k_{cat2}= 10 mM^{-2}h^{-1}, k_2= 0.1 mM^{-1}h^{-1} and **residence time τ = 1 h**.

Catalytic conditions: C1 < C2

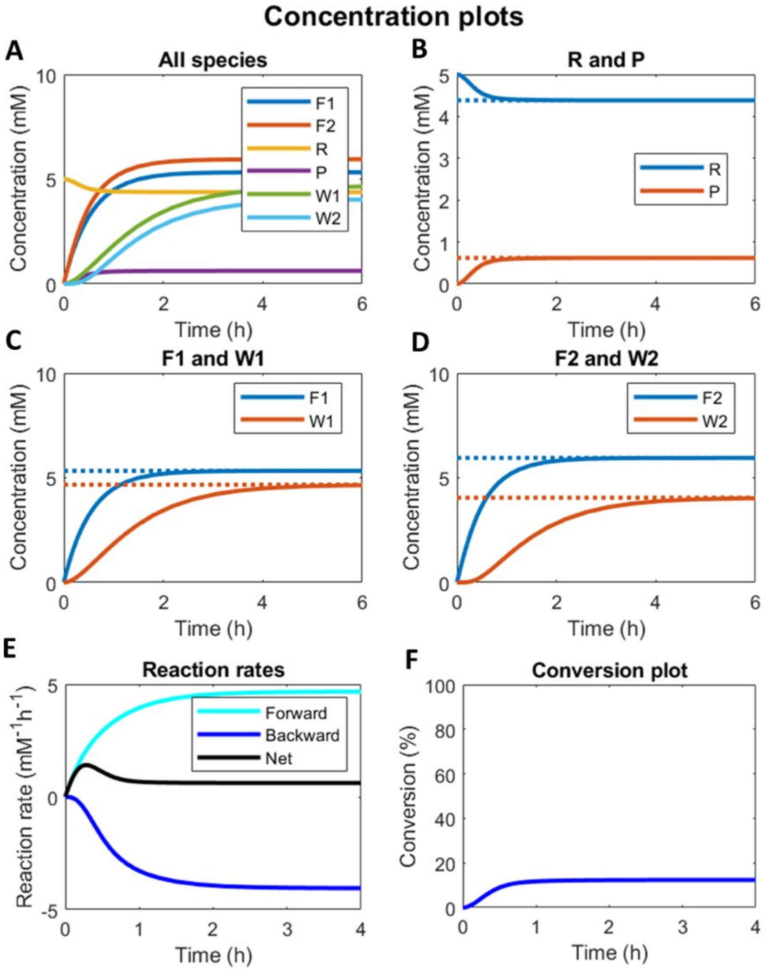

Figure S13: Numerical modelling output in CSTR: **(A).** Concentration of all species as a function of time. **(B).** Concentration of reactant R and product P as a function of time (solid lines) and their steady states (dashed lines). **(C).** Concentration of fuel F1 and waste W1 as a function of time (solid lines) and their steady states (dashed lines). **(D).** Concentration of reagent F2 and waste W2 as a function of time (solid lines) and their steady states (dashed lines). **(E).** Reaction rates for product P formation and degradation as a function of time. **(F).** Conversion plot of reactant R. Inlet conditions: [F1]=10 mM, [W1]=0 mM, [F2]=10 mM, [W2]=0 mM, [R]=5 mM, [P]=0 mM, **[C1]=0.01 mM, [C2]=0.1 mM**, rate constants k_{cat1}= 10 mM^{-2}h^{-1}, k_1= 0.1 mM^{-1}h^{-1}, k_{cat2}= 10 mM^{-2}h^{-1}, k_2= 0.1 mM^{-1}h^{-1} and **residence time τ = 1 h**.

Shorter residence time of 10 min

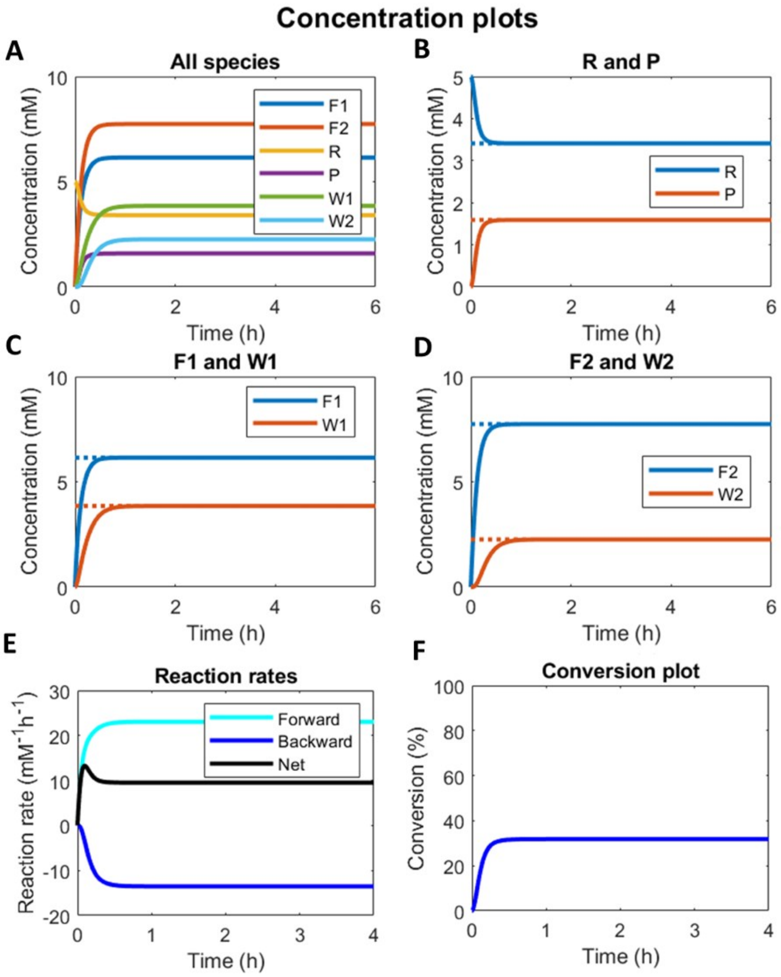

Figure S14: Numerical modelling output in CSTR: **(A).** Concentration of all species as a function of time. **(B).** Concentration of reactant R and product P as a function of time (solid lines) and their steady states (dashed lines). **(C).** Concentration of fuel F1 and waste W1 as a function of time (solid lines) and their steady states (dashed lines). **(D).** Concentration of reagent F2 and waste W2 as a function of time (solid lines) and their steady states (dashed lines). **(E).** Reaction rates for product P formation and degradation as a function of time. **(F).** Conversion plot of reactant R. Inlet conditions: [F1]=10 mM, [W1]=0 mM, [F2]=10 mM, [W2]=0 mM, [R]=5 mM, [P]=0 mM, **[C1]=0.1 mM, [C2]=0.1 mM**, rate constants k_{cat1}= 10 mM^{-2}h^{-1}, k_1= 0.1 mM^{-1}h^{-1}, k_{cat2}= 10 mM^{-2}h^{-1}, k_2= 0.1 mM^{-1}h^{-1} and **residence time τ = 10 min (0.167 h)**.

Longer residence time of 1.5 h

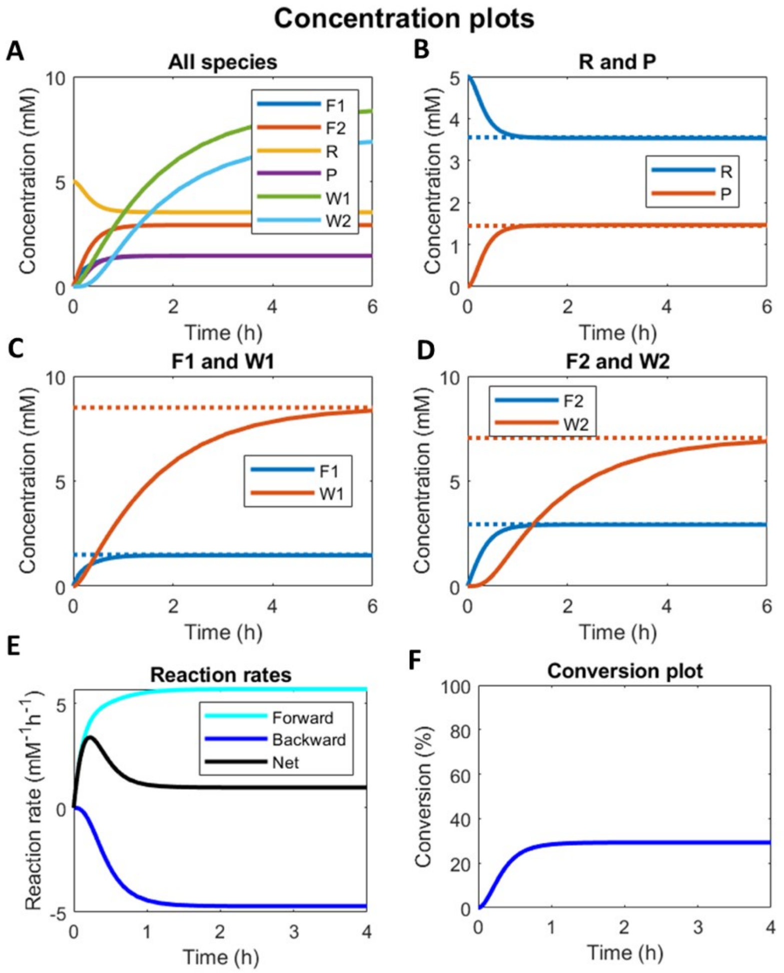

Figure S15: Numerical modelling output in CSTR: **(A).** Concentration of all species as a function of time. **(B).** Concentration of reactant R and product P as a function of time (solid lines) and their steady states (dashed lines). **(C).** Concentration of fuel F1 and waste W1 as a function of time (solid lines) and their steady states (dashed lines). **(D).** Concentration of reagent F2 and waste W2 as a function of time (solid lines) and their steady states (dashed lines). **(E).** Reaction rates for product P formation and degradation as a function of time. **(F).** Conversion plot of reactant R. Inlet conditions: [F1]=10 mM, [W1]=0 mM, [F2]=10 mM, [W2]=0 mM, [R]=5 mM, [P]=0 mM, **[C1]=0.1 mM, [C2]=0.1 mM**, rate constants k_{cat1}= 10 $mM^{-2}h^{-1}$, k_1= 0.1 $mM^{-1}h^{-1}$, k_{cat2}= 10 $mM^{-2}h^{-1}$, k_2= 0.1 $mM^{-1}h^{-1}$ and **residence time τ = 1.5 h**.
